# Gender Stereotypes in Science Education Resources: A Visual Content Analysis

**DOI:** 10.1371/journal.pone.0165037

**Published:** 2016-11-16

**Authors:** Anne H. Kerkhoven, Pedro Russo, Anne M. Land-Zandstra, Aayush Saxena, Frans J. Rodenburg

**Affiliations:** 1 Leiden Observatory, Leiden University, Leiden, The Netherlands; 2 Department of Science Communication and Society, Leiden University, Leiden, The Netherlands; 3 Department of Mathematics, Leiden University, Leiden, The Netherlands; University of Westminster, UNITED KINGDOM

## Abstract

More men are studying and working in science fields than women. This could be an effect of the prevalence of gender stereotypes (e.g., science is for men, not for women). Aside from the media and people’s social lives, such stereotypes can also occur in education. Ways in which stereotypes are visible in education include the use of gender-biased visuals, language, teaching methods, and teachers’ attitudes. The goal of this study was to determine whether science education resources for primary school contained gender-biased visuals. Specifically, the total number of men and women depicted, and the profession and activity of each person in the visuals were noted. The analysis showed that there were more men than women depicted with a science profession and that more women than men were depicted as teachers. This study shows that there is a stereotypical representation of men and women in online science education resources, highlighting the changes needed to create a balanced representation of men and women. Even if the stereotypical representation of men and women in science is a true reflection of the gender distribution in science, we should aim for a more balanced representation. Such a balance is an essential first step towards showing children that both men and women can do science, which will contribute to more gender-balanced science and technology fields.

## Introduction

The workforce in the fields of science, technology, engineering, and mathematics (hereafter called “STEM”) consists mainly of men; only 28.4% of employees in STEM are women (worldwide average of 2013 [1]). In other fields such as the humanities and education, employees are predominantly women [2]: in the US in 2014, 56.8% of employees in the humanities and arts and 75.9% of the employees in education are women [3]. The number of women working in STEM fields has increased with different amounts for several science professions (e.g., in 1960 27% of biologists were women, compared to 52.9% in 2008; 0.9% of engineers were women in 1960, compared to around 9.6% in 2008) [4]. Despite the increase of women in STEM fields, nearly all of those fields are still dominated by men. Attracting more women to work in the STEM fields is vital because their knowledge would otherwise form an untapped source. By including their views and ideas, more opportunities are created for achieving better research and innovation [2]. Society can make good use of more scientists in solving important issues in the present and future [5], such as developing drugs and techniques to cure diseases or developing technological innovations.

Carlos Moedas, European Union's Commissioner for Research & Innovation, gave a speech in 2014 highlighting the importance of reaching gender equality in STEM: “A diverse workforce, where women and minorities are represented, are simply more robust, flexible, more successful.” [6]. In the last years, the European Commission has been tackling this issue by funding several projects dealing with gender balance in science education (e.g., Science: It's a Girl Thing [7]). In order to equalize the number of men and women working in STEM fields it might be necessary to go back to early primary school (science) education. Research has shown that young girls are less interested in science and have fewer positive attitudes toward science than boys [8,9,10]. Gender stereotypes might be an underlying factor of why fewer girls than boys choose to study STEM courses in the first place [11]. Stereotypes are known to influence the interest development of an individual for the subject represented stereotypically [8]. An individual’s interest is higher when there is a positive correlation between what the stereotype represents and the individual’s self-image. A more positive correlation will show a higher preference for the subject represented [12]. Science is commonly aligned with masculinity, by both children and parents [13,14,15], and scientists are usually described or drawn as males [16,17,18]. Thus, stereotypic images of science and scientists can convey the message that science is for boys [19,20,13] and might present a negative correlation with the self-image of girls, causing girls to be less interested in science or in becoming a scientist. Moreover, gender stereotypic images of scientists can even directly affect boys’ and girls’ performance in science exercises [21]. For example, Spencer, Steele and Quinn (1998) [22] showed that girls’ performance in a math test was negatively influenced by simply telling them that boys do better on mathematics tests. In other words, due to gender stereotypes, girls can become less confident in their abilities regarding STEM subjects, resulting in fewer girls who choose STEM courses and careers.

Stereotypes develop due to complex socio-cultural factors [5]. These can differ depending on nationality, social status, age, etc. They shape people’s ideas about groups of people. With regard to gender differences, socio-cultural factors define typically male and typically female characteristics [23]. The main gender stereotypes show a division in the type of job: the job is either more scientific (male) or more nurturing (female). Common stereotypes are that men are more often scientists, engineers, or computer scientists, whereas women are often educators or work in health care [24].

In this study we focus on science education as a possible area where stereotypical images of STEM can originate but also where they can be counteracted. Stereotypic education materials and gender-biased teaching are important factors to consider when addressing gender stereotypes in science education. These aspects influence children starting at a young age and may cause them to acquire gender-biased ideas. Children’s stereotype consciousness increases rapidly between the ages of 6 and 10 [25]–thus, in primary school. Stereotyping may lead to gender-biased attitudes, students/children receiving gender-biased advice from teachers and parents concerning courses and study fields [2], and a socially driven lack of interest in STEM by girls since these fields are stereotypically more for boys [26].

Gender bias in primary school science education, the topic of the present study, can come about in multiple ways. First, teaching and assessment methods and teacher attitudes can be gender biased [27,28]. The teacher role in forming students' views on science is very important, as nearly half of the students in a study by Hutchinson, Stagg and Bentley (2009) [29] said their subject teacher is important for career advice. In addition to creating enthusiasm for science [30–32], the teacher plays a big part in creating gender-neutral or gender-balanced lessons and assessment methods [33]. *Gender neutrality* means that there is no distinction made between how boys and girls are taught or represented (e.g., in education resources). *Gender balance* means that the number of references or occurrences of men and women (e.g., in language or visuals) is equal. At present, there is still a gender bias, at least in the assessment of students’ physics tests, to the detriment of girls [34]. Combining teaching and assessment in a gender-neutral way and teaching in a gender-balanced way might be successful in raising girls’ interest in science. Such gender-inclusive teaching will help to match girls’ self-image of working in science (a negative image) with their actual capacities of working in science (positive—girls can do science as well as boys) [5]. Moreover, this approach to teaching will help to motivate and interest both genders. Female role models in the science classroom can also help to improve girls’ self-image about being able to do science [5].

A second manner in which gender bias could be brought into the classroom is by using gender biased science education resources. Gender stereotypes can be present in the context of education resources. The context can be gender biased by the manner in which different science topics are presented. For example, studies have compared different contexts of the same science subject and looked at whether boys and girls had a preference for a context. In a study by Kerger, Martin, and Brunner [8], it was found that girls became much more interested in different science subjects when they were introduced in a feminine context as opposed to a masculine context, which is the standard context of the science subjects. A feminine context would be “discuss the dangers of smoking,” and the masculine context would be “examine which poisons have an effect on the nervous system.” Despite the positive effect on girls’ interest, using only feminine contexts is to the detriment of boys [8]. Therefore, a mix of masculine and feminine contexts can be implemented in teaching materials or methods to make them more gender-balanced.

Third, gender bias can be present in education resources in the type of language and visual content. Visual content comprises images (drawings and pictures) and videos (film and animations). In a study by Lee and Collins [35], English language textbooks were investigated for both visual content and language. They found that men were depicted as working nearly twice as often as women and that women were portrayed as victims or caring more often than men. The results for language showed only a few instances of gender-biased language. In most instances, language was gender-neutral or both male/female versions of a word were used (e.g., he/she). Thus, the visual content was more gender-biased than the language. Gender bias in visual content can be investigated by determining the ratio of men and women in the visuals, as well as what role the characters play: are they stereotypes or not [36–38]? Another example of a study on visual content and language is that of Moser and Hannover [39]. In school textbooks for German language, they found a balance in the number of boys and girls depicted, but women were depicted less often than men. The same study also looked into the type of activity of each depicted person but found no significant differences in the type of activities between men and women.

In addition to school textbooks, teachers can nowadays make use of many education resources available online [40,41]. Since 2005, the installation of interactive whiteboards in the classroom has increased rapidly [41]. This enabled teachers to use online education resources in the classroom. From 2002 to 2005, there was an increase in primary school teachers using online resources in the classroom, from 10% to 38% [42]. A study from 2007 with beginning teachers found that many of them search the internet for online resources on a daily basis [43]. These online resources thus form an interesting and important group of teaching materials to investigate with regard to the presence of gender stereotypes.

The aim of the current study was to identify gender bias in the visual content of online science education resources. The visual content of children’s books, school textbooks, and education resources concerning men and women forms a direct portrait of men and women in society [38]. The manner in which men and women are portrayed can be an important factor influencing the development of children’s views of gender roles [12,21]. It is especially relevant to study the portrayal of men and women in education resources since they form the main source from which children acquire their ideas of science and scientists [44]. The visual content of education resources should, therefore, be gender-balanced in order to prevent a stereotypical view of the roles of men and women in society in general and in science in particular.

In the current study we investigated the visual content of online education resources in two online databases: Scientix and OERcommons, both providing resources for primary science education[45,46]. Scientix is a project that started in 2009 and is funded by the European Union. It forms a Europe-wide network for science teachers, researchers, and policy makers. The Scientix website is a platform for science education and provides over 2,200 science education resources, such as demonstrations, games, and experiments [31]. In the last three years of the project, Scientix has reached 365,300 visitors. Importantly, since Scientix is funded by the EU, it is very important to study the visual content of its science education resources. The impact of these resources will most likely take effect on a larger scale than locally developed and provided resources. The second database that was used is OERcommons. This is an international website launched in 2007, for open education resources, which means resources can be freely adapted, improved, and reused [46]. Since OERcommons is a very large database of education resources, it forms a good source to acquire an average sample of available resources to study for gender bias.

## Methods

### Sample

This was a content analysis study investigating the visual content of online science education resources. The study sample consisted of science education resources from the websites of Scientix and OERcommons with the following inclusion criteria: (1) primary school level (ages 4 to 11), (2) science field: astronomy, biology, chemistry, geology, mathematics, physics, and technology, (3) English language since there were many more resources in English than in another language on the two websites, and (4) format: doc, pdf, or html in order to conduct a language analysis (not described here). The total number of resources that met all four requirements was 2,164 resources. Since this is such a large number of resources to analyze, a required random sample size of 327 resources was calculated. The required random sample size was calculated for a population size of 2,164 resources, confidence level of 95%, margin of error of 5%, and response distribution of 50%. In the end, for the visual content analysis, 333 resources were analyzed.

### Analysis

The visual content analysis was conducted manually by one coder. Every resource was scanned for the presence of visual content. If there was visual content, each visual was checked for the number of men, women, boys, and girls, and the profession and activity of each person in the visual (see S1 Appendix 1 for the code books). The profession of each individual was studied to identify whether the number of men and women in science professions is equal. Additionally, the activity each person is doing was studied in order to identify whether a balance exists in the number of men and women in the more scientific and more nurturing activities. The same balance in type of activity was investigated for boys and girls.

**Fig 1:**
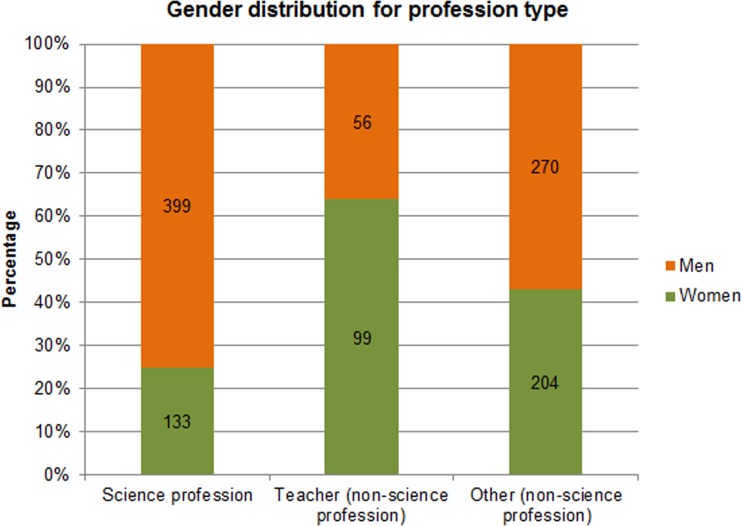
Gender distribution for profession type. N(people) = 1161.

**Fig 2:**
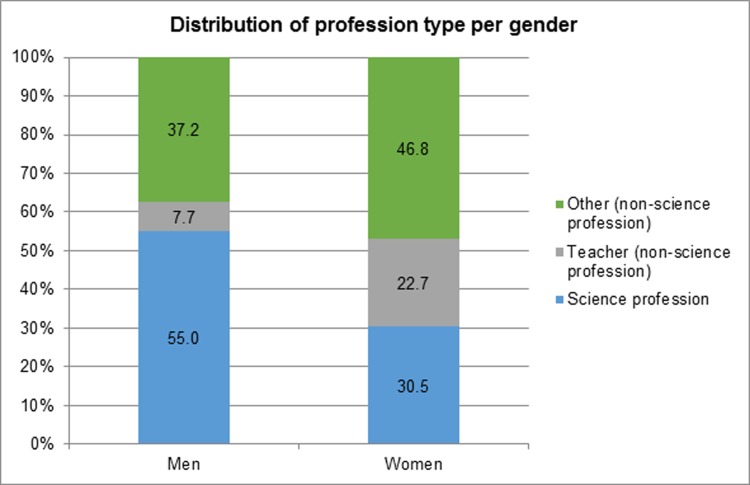
Distribution of profession type per gender. N(men) = 725; N(women) = 436; N(total) = 1161.

One extra coder was assigned to code the visual content of a random sample of 22 resources. In these resources, 240 depicted people were coded, which was more than 10% of the data used to draw conclusions on (i.e., 1,161 people, see Figs 1 and 2 in the Results section). This percentage should be sufficient according to Lombard, Snyder-Duch, and Bracken’s research [47]. The intercoder reliability was calculated by the method of Krippendorff’s alpha (K-alpha) [48]. For the categories teacher, science profession, and experiment, the intercoder reliability exceeded the lower limit for reliability (K-alpha = 0.70) for both genders; hence, conclusions were only drawn on those three categories. These include the most important category for this study (science profession) since the main question was whether the distribution of men and women was equal for the category science profession. For the activity category “teaching” no intercoder reliability was calculated because teaching was not applicable to the subset of the extra coder. The intercoder reliability test results for professions and activities are presented in S2 Appendix 2. Most of the disagreement between coders occurred on visuals for which it was difficult to obtain accurate counts, for example, in visuals with many people.

In order to compare the observed data with the expected data following the null hypothesis (the number of men and women in all types of professions and activities are equal), chi-squared tests were performed in the program R whereby the null hypothesis was rejected for *p* < 0.05 [49]. In this manner, it was possible to investigate whether there were any significant differences in the type of profession for each gender and in the type of activity for each gender.

## Results

In total, 3,191 depicted people were coded (in 333 resources). Boys were depicted most often (33.7%), followed by girls (29.9%), men (22.7%), and women (13.7%). Fig 1 shows the gender distribution for adults depicted as either in a science profession (e.g., an astronomer, technologist) or in a non-science profession (i.e., teacher or other non-science profession). The figure shows that there were many more men than women with a science profession (75.0% versus 25.0%, respectively), and fewer men than women were depicted as a teacher (36.1% versus 63.9%, respectively). There were slightly more men than women with an “other” type of profession (57.0% versus 43.0%, respectively).

A chi-squared test was performed on the three profession categories to identify whether the distribution of men and women in each category was significantly different. The test showed a significant difference for the gender distribution between the three profession types (χ^2^ (2, *N =* 1161) = 87.6085, *p* < 0.0001). From this, we concluded that there were significantly more men than women with a science profession and other profession, while there were significantly fewer men than women depicted as a teacher. However, there was a general overrepresentation of men in all of these visuals compared to women (62.4% versus 37.6%, respectively).

To answer the question whether there were still more men depicted with a science profession given the general overrepresentation of men, another chi-squared test was performed. This test compared the ratio of men and women depicted with a science profession (75% and 25%, respectively) and as a teacher (36.1% and 63.9%, respectively) to that of the third category (other non-science profession, 57% men and 43% women) because the latter presented a reference for the distribution of men and women in total in the studied resources. The chi-squared test (χ^2^ (1, *N* = 687) = 3.841) showed significant differences for science profession (p<0.001) and teacher (p<0.001). This means that, even after correcting for a general overrepresentation of men in the visuals, there were significantly more men than women depicted with a science profession, and significantly more women than men were depicted as teachers.

Fig 2 shows the distribution of profession type for each gender. In total, most men depicted in the science education resources were depicted with a science profession (55.0%) whereas 37.2% of men were depicted with an “other” type of profession. Very few men were depicted as teachers (7.7%). Most women were depicted with an “other” profession (46.8%), followed by a science profession (30.5%) and least often as a teacher (22.7%).

**Fig 3:**
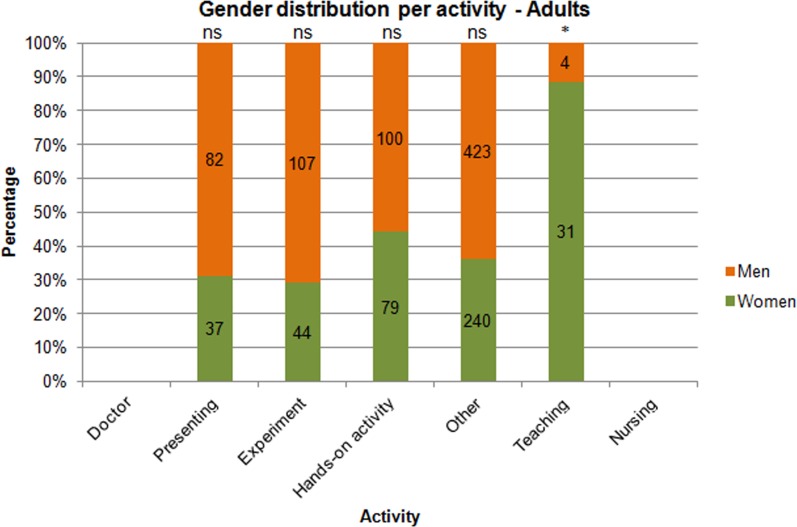
Gender distribution for activity type—Adults. N(people) = 1147. Note: * = p<0.001.

Fig 3 shows the gender distribution for activity type for adults. The activity categories were doctor, presenting, doing an experiment, doing a hands-on activity, teaching, nursing and “other” (this covered the people who could not be classified into the other categories). Fig 3 shows that there were more men than women in most categories (presenting, experiment, hands-on activity, and “other”) except for teaching, in which there were more women than men. The categories doctor and nursing held too few observations to perform statistical tests on and were therefore left out of the figure and further analysis. Fig 3 shows the absolute numbers of men and women. However, men are very highly overrepresented and, therefore, conclusions should be drawn on the corrected proportions of men.

A chi-squared test was performed to compare the ratio of men and women in experiment, presenting, and teaching to that of the summation of hands-on activity and “other” (χ^2^ (2, *N* = 305) = 5.991). The latter two categories represented a reference for the distribution of men and women in total in the studied resources. Therefore, chi-squared tests were performed only for experiment, presenting, and teaching, while correcting for the general overrepresentation of men. In this case, a result was significant when p< α/3 = 0.0167. We found a significant result for teaching (p<0.001), but not for experiment (p>0.02) and presenting (p>0.05). Thus, there seems to be an underrepresentation of men depicted as teaching.

**Fig 4:**
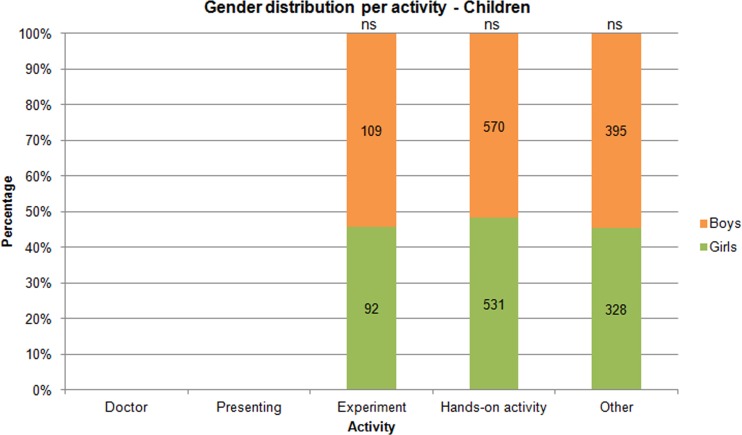
Gender distribution for activity type – Children. N(people) = 2025. The activity data for boys and girls were also tested with a chi-squared test, which showed no significant differences between the number of boys and girls in any activity category.

The figure below (Fig 4) shows the gender distribution for activity type for children. The activity categories were doctor, presenting, doing an experiment, doing a hands-on activity, and “other” (this covered the children who could not be classified into the other categories). Fig 4 shows that there were slightly more boys than girls in most categories (experiment, hands-on activity, and “other”). The categories doctor and presenting held too few observations and were therefore neglected from the figure and further analysis.

## Discussion

This study aimed to identify the status of online science education resources with regard to gender-biased visual content. Our investigation revealed that, in the online science education resources provided through the Scientix and OERcommons websites, women and men were portrayed in stereotypical ways while there was no statistically significant difference between boys and girls.

The visual content analysis showed that the largest proportion of people depicted were boys, followed, in decreasing numbers, by girls, men, and women. A similar pattern has been found in multiple studies over the last two decades. For example, this pattern is visible in German schoolbooks, as addressed by Moser and Hannover [39]. They studied visuals and language in German language and mathematics books and found that there were more men portrayed than women. Additionally, in the mathematics books, there were also more boys than girls. Two studies by Elgar [50,51] found that males were depicted in photographs more than four times as often as females in primary school books for science and science textbooks for secondary school. An older study [52] on Jamaican and British physics books for secondary school showed that more men than women were depicted, but for children an approximately equal number of boys and girls was found. It seems that different science textbooks show the same gender imbalance for adults but not for children.

With regard to the professions and activities of the men and women in the online science resources more men than women were depicted in science professions and more women than men were depicted as teachers. The latter result is similar to what Zittleman and Sadker [36] found when studying teacher education methods. They found that women were depicted as teachers twice as often as men in all textbooks they studied, including texts on science and on mathematics. Similarly to the profession outcomes, in activities, we found that there were more women than men performing the activity of teaching. However, the activity category ‘experiment’ was not significantly different in numbers of men and women. As for children, no significant differences between the number of boys and girls in any activity category in these resources have been found. Children, as opposed to adults, are thus presented in a gender balanced manner.

The present study shows no evidence of gender balance concerning adults in science and as scientists in online science education resources. Not only are men depicted more often than women in general in the studied resources, they are also depicted relatively more often in a science profession. Children, on the other hand, are depicted in a gender balanced manner with respect to types of activities, which is a positive sign and might help girls see themselves as capable of doing science. However, the adults in pictures and videos may serve as role models, making it very important that those visuals are also gender balanced [12,21,38,44]. Accordingly, we can conclude that there is already a gender imbalance in the visuals of science education resources for children at primary school level. The stereotyping of men in science and women in teaching is, thus, already present at this very early level of education. The earlier this imbalance is presented to children, the more impact it may have on them. At least for girls aged 5 to 7, stereotypes already affect their math test results [53]. Therefore, gender stereotypes in science education resources should be counteracted.

In order to counteract gender stereotypes in science education resources, it is important that the number of men and women in the visual content of educational resources is balanced, as well as the number of boys and girls. In this manner, girls can see that science is for women as well as for men. In addition, the type of professions and activities of men and women should be portrayed equally, as well as that of boys and girls. Related to this is to balance the number of people mentioned in texts, with regard to gender. In this way, stories in science education resources about boy characters and girl characters will be balanced. Another important way to counteract gender stereotypes in science education resources is by using gender-balanced language (e.g., not using the word “man” to describe all people, but using “humanity” or “people” instead) [54].

Future research should explore whether there are any differences between different science fields (e.g., astronomy, biology, chemistry, geology, mathematics, physics, and technology) with regard to the gender distribution for professions and activities in education resources. Since the distribution of men and women is different in different science fields [55], it can be expected that science education resources from the fields where there are many more men than women show a bigger gender imbalance than those of science fields where there is an equal distribution of men and women. Testing this will, therefore, offer two main insights: 1) which fields need to do much work to achieve more gender-balanced education resources about those science fields and 2) which fields already have relatively gender-neutral education resources. It is expected that resources about biology, for example, are less gender-biased than those about physics or astronomy (based on the distribution of men and women studying or working in those fields [56,57]).

In relation to the stereotype of scientists being male, an interesting opportunity for future research is to study scientist stereotyping in education resources. A world-wide occurring stereotype, at least among children, is that of the scientist [57]. When children are asked to draw a scientist, mostly they draw a middle-aged bald man wearing glasses and a white lab coat [17,18]. This stereotypical view of a scientist may be caused, at least in part, by stereotypical visuals in science education resources. This stereotyping may cause a low interest in girls for science and may cause them to view science as something more for boys. It would be interesting to explore this topic and to determine whether this phenomenon is present in the same abundance in science education resources for different age groups. This could show at what age this science stereotyping begins and, thus, when the influence on the children starts.

In conclusion, we found that in the studied online science education resources, women and men are portrayed in stereotypic ways, with more men depicted with science professions and more women depicted as teachers. Even if this is a true reflection of the distribution of men and women in those professions, we should aim for a more balanced representation so girls and boys see examples of both female and male scientists. We believe that a more balanced view will help girls and boys to make their own decisions concerning their studies and professions rather than those decisions being influenced by gender stereotypes.

## Supporting Information

S1 Appendix Visual content code books. (PDF)

S2 Appendix Intercoder reliability results. (PDF)
