# Supplementary material for: Gender Stereotypes in Science Education Resources: A Visual Content Analysis
Source: PLoS One. 2016 Nov 16;11(11):e0165037. doi: 10.1371/journal.pone.0165037 (PMC5112807; doi:10.1371/journal.pone.0165037)
Supplement: S1 Appendix — (PDF) [file pone.0165037.s001.pdf]

Appendix 1 – Visual content code books

Table 5. Code book for identifying different professions of people depicted in the visuals of science education resources, based on [58] and [37].

Profession	
-	Other (non-science profession)
1	Teacher primary school
2	Teacher secondary school
3	Astronomer
4	Biologist
5	Chemist
6	Geologist
7	Mathematician
8	Physicist
9	Technologist
10	Presenter
11	Scientist
12	Student
13	Doctor
14	Astronaut

Table 6. Code book for identifying different professions (pooled categories) of people depicted in the visuals of science education resources, based on [58] and [37].

Profession	
Other (non-science profession)	Other (non-science profession)<br>Presenter
Teacher	Teacher primary school<br>Teacher secondary school
Science profession	Astronaut<br>Astronomer<br>Biologist<br>Chemist<br>Doctor<br>Geologist<br>Mathematician<br>Physicist<br>Technologist<br>Scientist
Student	Student

Table 7. Code book for identifying different activities of people depicted in the visuals of science education resources, based on [58] and [37].

Activity	
0	Other than any below: Other
1	Teaching
2	Art activity
3	Astronomy experiment
4	Astronomy activity
5	Biology experiment
6	Biology activity
7	Chemistry experiment
8	Chemistry activity
9	Geology experiment
10	Geology activity
11	Mathematics activity
12	Physics experiment
13	Physics activity
14	Technology experiment
15	Technology activity
16	Presenting
17	Nursing
18	Doctor

Table 8. Code book for identifying different activities (pooled categories) of people depicted in the visuals of science education resources, based on [58] and [37].

Activity	
Other	Other
Experiment	Astronomy experiment<br>Biology experiment<br>Chemistry experiment<br>Geology experiment<br>Physics experiment<br>Technology experiment
Hands-on activity	Art activity<br>Astronomy activity<br>Biology activity<br>Chemistry activity<br>Geology activity<br>Mathematics activity<br>Physics activity<br>Technology activity
Teaching	Teaching
Nursing	Nursing
Doctor	Doctor
Presenting	Presenting

References

37. Blumberg RL. The invisible obstacle to educational equality: Gender bias in textbooks. Prospects. 2008;38(3):345-61.

58. Bell P. Content analysis of visual images. In: Van Leeuwen T, Jewitt C. The handbook of visual analysis. London, UK: SAGE Publications Ltd; 2004. p. 10-35. Available: http://srmo.sagepub.com/view/the-handbook-of-visual-analysis/n2.xml.
