# Supplementary material for: Gender Stereotypes in Science Education Resources: A Visual Content Analysis
Source: PLoS One. 2016 Nov 16;11(11):e0165037. doi: 10.1371/journal.pone.0165037 (PMC5112807; doi:10.1371/journal.pone.0165037)
Supplement: S2 Appendix — (PDF) [file pone.0165037.s002.pdf]

Appendix 2 – Intercoder reliability results

Table 9. Intercoder reliability results for professions with Krippendorff's alpha.

Profession	Men	Women	Boys	Girls
Other (non-science profession)	0.439	0.246	0.454	0.537
Teacher	0.778[a]	0.833[a]	- [b]	- [b]
Science profession	0.803[a]	0.872[a]	- [b]	- [b]
Student	- [b]	- [b]	0.729[a]	0.810[a]

[a] **Variable is reliable (K-alpha > 0.70)**

[b] **No people were coded for this category in the subsample of the second coder**

Table 10. Intercoder reliability results for activities with Krippendorff's alpha.

Activity	Men	Women	Boys	Girls
Other	0.415	0.356	0.244	0.464
Teaching	- [b]	- [b]	- [b]	- [b]
Hands-on activity	0.369	0.334	0.119	0.368
Nursing	0	- [b]	- [b]	- [b]
Experiment	0.828[a]	0.835[a]	0.181	-0.151
Presenting	0.679	0.833[a]	-0.024	-0.049

[a] **Variable is reliable (K-alpha > 0.70)**

[b] **No people were coded for this category in the subsample of the second coder**
